# The HIV Care Continuum: Changes over Time in Retention in Care and Viral Suppression

**DOI:** 10.1371/journal.pone.0129376

**Published:** 2015-06-18

**Authors:** Baligh R. Yehia, Alisa J. Stephens-Shields, John A. Fleishman, Stephen A. Berry, Allison L. Agwu, Joshua P. Metlay, Richard D. Moore, W. Christopher Mathews, Ank Nijhawan, Richard Rutstein, Aditya H. Gaur, Kelly A. Gebo, HIV Research Network

**Affiliations:** 1 Department of Medicine, Perelman School of Medicine, University of Pennsylvania, Philadelphia, PA, United States of America; 2 Leonard Davis Institute of Health Economics, University of Pennsylvania, Philadelphia, PA, United States of America; 3 Center for Biostatistics and Epidemiology, Perelman School of Medicine, University of Pennsylvania, Philadelphia, PA, United States of America; 4 Center for Financing, Access, and Cost Trends, Agency for Healthcare Research and Quality, Rockville, MD, United States of America; 5 Department of Medicine, Johns Hopkins University School of Medicine, Baltimore, MD, United States of America; 6 General Medicine Division, Massachusetts General Hospital, Boston, MA, United States of America; 7 Department of Medicine, University of California San Diego, San Diego, CA, United States of America; 8 Department of Medicine, University of Texas Southwestern, Dallas, TX, United States of America; 9 Division of General Pediatrics, Children's Hospital of Philadelphia, Philadelphia, PA, United States of America; 10 Department of Infectious Diseases, St. Jude’s Children's Hospital, Memphis, TN, United States of America; University of Athens, Medical School, GREECE

## Abstract

**Background:**

The HIV care continuum (diagnosis, linkage to care, retention in care, receipt of antiretroviral therapy (ART), viral suppression) has been used to identify opportunities for improving the delivery of HIV care. Continuum steps are typically calculated in a conditional manner, with the number of persons completing the prior step serving as the base population for the next step. This approach may underestimate the prevalence of viral suppression by excluding patients who are suppressed but do not meet standard definitions of retention in care. Understanding how retention in care and viral suppression interact and change over time may improve our ability to intervene on these steps in the continuum.

**Methods:**

We followed 17,140 patients at 11 U.S. HIV clinics between 2010-2012. For each calendar year, patients were classified into one of five categories: (1) retained/suppressed, (2) retained/not-suppressed, (3) not-retained/suppressed, (4) not-retained/not-suppressed, and (5) lost to follow-up (for calendar years 2011 and 2012 only). Retained individuals were those completing ≥2 HIV medical visits separated by ≥90 days in the year. Persons not retained completed ≥1 HIV medical visit during the year, but did not meet the retention definition. Persons lost to follow-up had no HIV medical visits in the year. HIV viral suppression was defined as HIV-1 RNA ≤200 copies/mL at the last measure in the year. Multinomial logistic regression was used to determine the probability of patients’ transitioning between retention/suppression categories from 2010 to 2011 and 2010 to 2012, adjusting for age, sex, race/ethnicity, HIV risk factor, insurance status, CD4 count, and use of ART.

**Results:**

Overall, 65.8% of patients were retained/suppressed, 17.4% retained/not-suppressed, 10.0% not-retained/suppressed, and 6.8% not-retained/not-suppressed in 2010. 59.5% of patients maintained the same status in 2011 (kappa=0.458) and 53.3% maintained the same status in 2012 (kappa=0.437).

**Conclusions:**

Not counting patients not-retained/suppressed as virally suppressed, as is commonly done in the HIV care continuum, underestimated the proportion suppressed by 13%. Applying the care continuum in a longitudinal manner will enhance its utility.

## Introduction

Advances in the treatment of HIV infection have led to the development of effective, more convenient antiretroviral therapy (ART).[1,2] Better therapies have improved adherence to ART, reduced HIV-related complications, and increased survival.[3–7] However, to benefit fully from ART, HIV-infected individuals must fulfill several steps along the HIV care continuum—HIV diagnosis, linkage to care, retention in care, receipt of ART, and HIV viral suppression.[8,9] Yet in the United States (U.S.), only 25% of HIV-infected adults complete all of these steps.[8]

The HIV care continuum has been used by federal, state, and local agencies to identify gaps and opportunities for improving the delivery of HIV care.[8–11] While the continuum is a useful framework for monitoring HIV care, it has a number of limitations. First, continuum steps are typically calculated in a conditional manner, with the number of persons completing the prior step serving as the base population for the next step. This may not be problematic for early stages in the continuum but may be an issue for the final steps of retention in care and viral suppression. Specifically, this approach may underestimate the prevalence of viral suppression by excluding patients who are suppressed but do not meet standard definitions of retention in care; few studies have estimated the size of this group.[12,13] Second, the continuum is largely static, providing a snapshot of HIV testing, engagement in care, and viral suppression at a specific time point or during a set time period. Third, the continuum focuses on populations, not individuals. As such, data are presented in the aggregate and may not accurately capture changes across time for individual patients.

Retention in care is associated with HIV viral suppression,[14] but the proportion of patients remaining retained and suppressed over time is not known. Understanding how individuals move between states of retention/non-retention and suppression/non-suppression over time may improve our ability to intervene on these steps in the continuum. Using data from a large, U.S. multisite cohort, we followed individual patients over a three-year period to determine how retention in care and viral suppression statuses interact and change over time.

## Methods

### Study Sample and Data Collection

We analyzed prospectively collected data from the HIV Research Network (HIVRN), a consortium of clinics that provide care to HIV-infected patients.[15] All patients presenting for care were offered enrollment in the HIVRN, excluding one-time consultations and incarcerated individuals. Data were abstracted from medical records at each site and sent to a data coordinating center after removing personal identifying information. After quality control and verification, data were combined across sites to produce a uniform database. Institutional review boards (IRBs) at each site (complete list of sites can be found in supplemental file 1) and the data coordinating center at Johns Hopkins University approved the collection and analyses of these data. IRBs at some clinics required written informed consents, while others waived the requirement because only existing anonymized and de-identified data were collected.

Data from 11 HIVRN sites, located in the Northeastern (5), Midwestern (1), Southern (2), and Western (3) sections of the U.S., were included in analyses. Adult patients (age ≥18 years) with at least one primary HIV outpatient visit and one HIV-1 RNA level between January 1 and December 31, 2010 were eligible for inclusion. Patients newly enrolled at HIVRN sites between July 1 and December 31, 2010 were excluded, as they did not provide adequate time to measure retention in care. Eligible patients were followed at these 11 HIVRN sites through December 31, 2012. Patients who died, transferred their care outside of the HIVRN, and who had no recorded viral load tests after 2010 were excluded from subsequent years’ analyses.

### Definitions of Variables

Retention in care was based on the U.S. *National HIV/AIDS Strategy* metric.[16] Individuals “retained in care” were those completing 2 or more HIV medical visits separated by ≥90 days in a calendar year. Persons “not retained in care” completed at least one HIV medical visit during the calendar year, but did not meet the retention definition (e.g. only attended one visit or attended multiple visits within a single 90-day period). These individuals were distinguished from patients lost to follow-up (LTFU), who had no HIV medical visits in a calendar year. HIV viral suppression was categorized as suppressed (HIV-1 RNA ≤ 200 copies/mL) and not suppressed (HIV-1 RNA > 200 copies/mL) at the last measure in the calendar year.

For each patient, age as of January 1, 2010 was divided into four groups: 18–29, 30–39, 40–49, and over 50 years old. Race/ethnicity was categorized as non-Hispanic White, non-Hispanic Black, Hispanic, and other/unknown. HIV transmission risk factor was grouped into men who had sex with men (MSM), heterosexual transmission (HET), injection drug use (IDU), and other/unknown. Patients who had IDU in combination with another risk factor (e.g. MSM, heterosexual transmission) were classified as IDU. Insurance coverage in 2010 was categorized as private, Medicaid, Medicare (including dual eligibles), uninsured, or other/unknown. Patients whose care was funded by Ryan White, those recorded as self-pay, and those covered by local governmental programs were classified as uninsured. First CD4 count recorded in 2010 was grouped as ≤ 200, 201–350, 351–500, > 500 cells/mm^3^, and missing. Use of ART was defined as receiving 3 antiretroviral drugs from two or more classes for ≥ 6 months in 2010.

### Statistical Analyses

Standard descriptive analyses of demographic and clinical characteristics of the sample were conducted. For each calendar year, patients were classified into one of five categories: (1) retained in care and virally suppressed (retained/suppressed), (2) retained in care and not virally suppressed (retained/not-suppressed), (3) not retained in care and virally suppressed (not-retained/suppressed), (4) not retained in care and not virally suppressed (not-retained/not-suppressed), and (5) LFTU (for calendar years 2011 and 2012 only). Patients LTFU in 2011 could re-enter the analysis for 2012 if they had a HIV medical visit in 2012. Retention/suppression status in 2010 was cross-classified with retention/suppression status in 2011 to observe one-year transitions, and with retention/suppression status in 2012 to observe two-year transitions. Kappa statistics were calculated to assess the agreement in retention/suppression classifications over time (2010 to 2011 and 2010 to 2012).[17]

Multinomial logistic regression was used to determine the probability of patients’ transitioning between the retention/suppression categories from 2010 to 2011 and 2010 to 2012, adjusting for differences in age, sex, race/ethnicity, HIV risk factor, insurance status, CD4 count, and use of ART. To account for differences across sites, we included indicator variables for each site. Based on regression results, we calculated marginal predicted probabilities for transitioning from one retention/suppression category to another over 1-year and 2-year periods.[18] Statistical analyses were performed using Stata 12.1 (Stata Corporation, College Station, TX).

## Results

A total of 17,140 eligible adults were in care at HIVRN clinics in 2010 (Table 1). In that year, most patients were male (73.6%), between 40–49 years old (38.4%), racial/ethnic minorities (67.1%), and had Medicaid (34.0%) or no health insurance (27.9%); 34.8% had a first CD4 count ≤350 cells/mm^3^ and 79.1% were on ART. Overall, 65.3% of patients were retained/suppressed, 17.7% were retained/not-suppressed, 10.0% were not-retained/suppressed, and 6.9% were not-retained/not-suppressed in 2010. The HIV care continuum would typically not count patients in the not-retained/suppressed group, who comprised 13% of all individuals achieving viral suppression, as virally suppressed. Younger individuals, females, blacks, those with IDU or MSM risk, individuals with Medicaid or no insurance, persons with lower CD4 counts, and those not receiving ART were relatively less likely to be retained/suppressed compared to their respective counterparts (*P* <0.001). (Table 1).

**Table 1:** Demographic and Clinical Characteristics of the Sample and Association with Retention/Suppression Status in 2010.

Characteristics		Total N = 17,140 (%)^a^	R/S (N = 11,194)^b^	R/NS (N = 3,035)^b^	NR/S (N = 1,720)^b^	NR/NS (N = 1,191)^b^
**Age (years) in 2010**						
	18–29	1,547 (9.0)	48.7%	26.5%	10.8%	14.0%
	30–39	3,195 (18.6)	59.5%	20.4%	11.1%	9.0%
	40–49	6,581 (38.4)	65.1%	17.4%	10.6%	7.0%
	≥ 50	5,817 (33.9)	73.1%	14.2%	8.7%	4.0%
**Sex**						
	Male	12,620 (73.6)	66.6%	16.5%	10.3%	6.6%
	Female	4,520 (26.4)	61.8%	21.2%	9.2%	7.8%
**Race/Ethnicity**						
	White	5,249 (30.6)	69.3%	11.9%	13.2%	5.5%
	Black	7,756 (45.3)	61.0%	22.0%	8.5%	8.5%
	Hispanic	3,730 (21.8)	68.8%	17.0%	8.5%	5.8%
	Other/Unknown	405 (2.4)	64.0%	17.3%	12.8%	5.9%
**HIV Risk Factor**						
	MSM	6,207 (36.2)	63.1%	20.0%	9.3%	7.6%
	Heterosexual	7,796 (45.5)	68.7%	14.9%	10.5%	5.9%
	IDU	2,656 (15.5)	62.5%	19.5%	9.9%	8.2%
	Other/Unknown	481 (2.8)	55.1%	23.1%	13.7%	8.1%
**Insurance in 2010**						
	Private	2,644 (15.4)	70.2%	12.2%	12.6%	5.1%
	Medicaid	5,823 (34.0)	63.4%	22.1%	7.3%	7.2%
	Medicare	3,324 (19.4)	69.4%	16.1%	8.8%	5.7%
	Ryan White/Uninsured	4,773 (27.9)	53.1%	16.6%	11.9%	8.3%
	Other/Unknown	576 (3.4)	56.9%	16.8%	17.2%	9.0%
**First CD4 Count in 2010**						
	≤ 200 cell/mm^3^	2,749 (16.0)	45.8%	33.2%	7.0%	14.0%
	201–350 cell/mm^3^	3,228 (18.8)	63.5%	19.3%	9.1%	8.1%
	351–500 cell/mm^3^	3,787 (22.1)	67.5%	15.7%	10.5%	6.4%
	> 500 cell/mm^3^	7,296 (42.6)	72.6%	12.3%	11.3%	3.9%
	Missing	80 (0.5)	38.8%	13.8%	20.0%	27.5%
**Use of ART in 2010**						
	No	3,573 (20.9)	10.5%	56.5%	4.8%	28.2%
	Yes	13,567 (79.1)	70.1%	14.3%	10.5%	5.1%

**Abbreviations:** ART, antiretroviral therapy; HET, heterosexual transmission; HIV, human immunodeficiency virus; IDU, injection drug use; MSM, men who have sex with men; R = retained; S = suppressed (virologically); NR = not retained; NS = not suppressed (virologically).

All associations are statistically significant, p<0.001.

^a^ Column percentages.

^b^ Row percentages.

Tables 2 and 3 presents observed transitions in retention/suppression status between calendar years 2010–2011 and 2010–2012. For analysis of retention/suppression in 2010–2011, patients who died (n = 175), transferred their care outside of the HIVRN (n = 107), and who had no recorded viral load test (n = 483) were excluded, leaving a sample of 16,375. Analysis of retention/suppression in 2010–2012 excluded those who died or transferred their care in 2011 (n = 282), died in 2012 (n = 173), transferred their care in 2012 (n = 40), or had no recorded viral load test in 2012 (n = 407), leaving 16,238 observations. In total, 59.5% of patients maintained the same retention/suppression status in 2011 as in 2010 (kappa = 0.458) and 53.3% maintained the same status in 2012 as in 2010 (kappa = 0.437). In multinomial logistic regression models (Tables A and B in S1 File), the pattern of predicted transitions in retention/suppression status over time remained similar to the unadjusted results (Tables 4 and 5).

**Table 2:** Retention/Suppression Status in Calendar-Years 2011 by Retention/Suppression Status in Calendar-Year 2010.

		Status in 2011				
	Group	R/S N = 10,158	R/NS N = 2,060	NR/S N = 1,165	NR/NS N = 606	LTFU N = 2,386
**Status in 2010**	**R/S N = 10,770**	8,261 (76.7%)	667 (6.2%)	800 (7.4%)	136 (1.3%)	906 (8.4%)
	**R/NS N = 2,848**	894 (31.3%)	1,113 (39.1%)	99 (3.5%)	262 (9.2%)	480 (16.9%)
	**NR/S N = 1,638**	812 (49.6%)	78 (4.8%)	206 (12.6%)	52 (3.2%)	490 (29.9%)
	**NR/NS N = 1,119**	191 (17.1%)	202 (18.1%)	60 (5.4%)	156 (13.9%)	510 (45.6%)

**Abbreviations:** R = retained; S = suppressed (virologically); NR = not retained; NS = not suppressed (virologically); LTFU = loss to follow-up. **Note:** N = 16,375, excludes patients who transferred care (n = 107), died (n = 175), and with no viral load measure (n = 483) in 2011. Chi-square (12 df) = 5,400; P <0.001. Kappa = 0.458.

**Table 3:** Retention/Suppression Status in Calendar-Years 2012 by Retention/Suppression Status in Calendar-Year 2010.

		Status in 2012				
	Group	R/S N = 9,636	R/NS N = 1,547	NR/S N = 1,246	NR/NS N = 471	LFTU N = 3,338
**Status in 2010**	**R/S N = 10,669**	7,654 (71.7%)	603 (5.7%)	823 (7.7%)	141 (1.3%)	1,448 (13.6%)
	**R/NS N = 2,833**	1,033 (35.5%)	714 (25.2%)	143 (5.1%)	195 (6.9%)	748 (26.4%)
	**NR/S N = 1,619**	724 (44.7%)	79 (4.9%)	206 (12.7%)	48 (3.0%)	562 (34.7%)
	**NR/NS N = 1,117**	225 (20.1%)	151 (13.5%)	74 (6.6%)	87 (7.8%)	580 (51.9%)

**Abbreviations:** R = retained; S = suppressed (virologically); NR = not retained; NS = not suppressed (virologically); LTFU = loss to follow-up. **Note:** N = 16,238, excludes the 175 patients who died in 2011 and patients who transferred care (n = 173), died (n = 40), and with no viral load measure (n = 407) in 2012. Chi-square (12 df) = 3,200; P <0.001. Kappa = 0.437.

**Table 4:** Predicted Probabilities for Retention in Care and Viral Suppression Status in Calendar-Years 2011 by Retention/Suppression Status in Calendar-Year 2010, from Multinomial Regression Models.

		Status in 2011				
	Group	R/S	R/NS	NR/S	NR/NS	LTFU
**Status in 2010**	**R/S**	74.7%	7.4%	7.1%	1.5%	9.3%
	**R/NS**	40.3%	31.9%	5.0%	7.5%	14.6%
	**NR/S**	41.9%	4.9%	12.1%	3.6%	37.5%
	**NR/NS**	24.1%	14.6%	7.5%	9.7%	44.0%

**Abbreviations:** R = retained; S = suppressed (virologically); NR = not retained; NS = not suppressed (virologically); LTFU = loss to follow-up.

**Table 5:** Predicted Probabilities for Retention in Care and Viral Suppression Status in Calendar-Years 2012 by Retention/Suppression Status in Calendar-Year 2010, from Multinomial Regression Models.

		Status in 2012				
	Group	R/S	R/NS	NR/S	NR/NS	LTFU
**Status in 2010**	**R/S**	70.0%	6.4%	7.6%	1.0%	14.5%
	**R/NS**	43.5%	20.7%	6.1%	6.0%	23.7%
	**NR/S**	38.3%	4.7%	12.8%	3.2%	41.0%
	**NR/NS**	26.5%	11.1%	7.8%	6.2%	48.5%

**Abbreviations:** R = retained; S = suppressed (virologically); NR = not retained; NS = not suppressed (virologically); LTFU = loss to follow-up.

There were wide variations in individuals’ future retention/suppression status based on their original classification in 2010. Those retained/suppressed in 2010 were relatively stable, with 76.7% remaining in the same group in 2011 and 71.7% in 2012. In contrast, relatively few individuals not-retained/not-suppressed in 2010 remained in the same category in 2011 (13.9%) and 2012 (7.8%). Approximately half of individuals not-retained/not-suppressed in 2010 were subsequently LTFU in 2012, and one-fifth transitioned to the retained/suppressed category in 2012.

A high proportion (44.7–49.6%) of patients not-retained/suppressed in 2010 moved to the retained/suppressed category in subsequent years; 12.6–12.7% remained unchanged and 29.9–34.7% were LFTU. Of those retained/not-suppressed in 2010, 39.1% and 25.2% remained in the same category in 2011 and 2012, respectively. However, over 30% moved to retained/suppressed and between 16.9–26.4% were LTFU in subsequent years.

Over the study period, 1.7% of persons retained/suppressed died, compared to 3.2% of retained/not suppressed, 1.5% of not-retained/suppressed, and 2.7% of those not-retained/not-suppressed.

## Discussion

These results highlight certain patient experiences currently not captured by the HIV care continuum. First, many individuals had transitions in their retention/suppression status over time, with only 60% of patients maintaining the same status over a 1-year period and 53% over a 2-year period. Fortunately, patients retained/suppressed in 2010 were the most stable group. Second, excluding patients who did not meet standard definitions of retention in care underestimated the proportion virally suppressed by 13%. Understanding how individuals engage in outpatient care over time and developing monitoring systems to better capture these experiences is essential to improving HIV outcomes and care delivery.

Transitions from one retention/suppression category to another were common, especially for individuals not-retained/suppressed, retained/not-suppressed, and not-retained/not-suppressed. Among those virally suppressed, approximately 20–25% had viral failure or were LTFU in subsequent years. Conversely, approximately one-third of patients not-retained and/or not-suppressed moved into the retained/suppressed group over time. These transitions are likely influenced by multiple factors at the patient (e.g. health literacy, co-morbid conditions, support system, ART adherence), clinic/health system (e.g. patient-provider relationship, co-location of multiple services, ART receipt), and environmental (e.g. competing life activities, distance to clinic) levels.[19,20]

Individuals virally suppressed but not retained in care are commonly counted as not suppressed in the HIV care continuum. These patients may be receiving care at multiple clinics, transitioning from one clinic to another, or newly incarcerated, and thus may not meet retention criteria at each individual site of care. For example, among 12,759 HIV-infected adults seen at Ryan White-funded clinics in Philadelphia, PA in 2008–2010, 8% received care at more than one clinic.[21] Alternatively, this group may represent persons with excellent self-management skills.[22–24] HIV treatment guidelines now recommend ART for all HIV-infected individuals, regardless of CD4 count, and less frequent laboratory monitoring in adherent patients with a suppressed viral load and stable immunologic status.[25] As the proportion of HIV-infected individuals on ART increases, the number of persons who are not-retained/suppressed is likely to grow. Moreover, less frequent laboratory monitoring may translate to fewer outpatient visits among the subset of patients with well-controlled HIV. These changes underscore the importance of counting not-retained/suppressed individuals as suppressed when monitoring the HIV care continuum. Lastly, this not-retained/suppressed group may represent individuals with work and/or family conflicts or inconvenient care (e.g. long wait times, lengthy travel time) that result in missed clinic appointments but not compromised medication adherence.[26] Additional data are needed to better understand adherence practices and long-term outcomes of this group.

This study is limited by its retrospective nature and inability to capture visits to clinics outside the HIVRN. It is possible that the HIV-1 RNA measure could have occurred relatively early in the calendar year; thus failure to be retained in care may have occurred after viral load measurement. Results may vary depending on the definition of retention in care used; yet, multiple studies have shown that retention measures are moderately correlated.[27,28] It is possible that clinical factors (e.g., CD4 count) at ART initiation may affect subsequent retention. However, this study did not use an ART-naive cohort and could not examine this possibility. Additional research evaluating associations between patient factors at time of first ART prescription and the outcomes is needed. Although HIVRN sites encompass a broad geographic distribution and include patients with a variety of demographic and clinical characteristics, our findings may not generalize to all HIV-infected patients in care but rather to those in care at large, urban HIV clinics with highly experienced providers.

The HIV care continuum is a helpful framework for monitoring HIV care. However, applying it in a longitudinal framework will enhance its utility. Strictly requiring retention in care criteria be met in order to consider individuals virally suppressed may underestimate the proportion of patients achieving viral suppression. These findings have important implications for monitoring the quality of HIV care and for meeting targets established by the U.S. National HIV/AIDS Strategy.[29,30] A better understanding of how HIV-infected persons use outpatient HIV services is essential to improving the assessment and design of HIV care delivery.

## Supporting Information

S1 File Multivariate Multinomial Logistic Regression Model of Retention in Care and Viral Suppression. (Table A) Multivariate Multinomial Logistic Regression Model of Retention in Care and Viral Suppression, 2010–2011. (Table B) Multivariate Multinomial Logistic Regression Model of Retention in Care and Viral Suppression, 2010–2012.
(DOCX)
