# Supplementary material for: The HIV Care Continuum: Changes over Time in Retention in Care and Viral Suppression
Source: PLoS One. 2015 Jun 18;10(6):e0129376. doi: 10.1371/journal.pone.0129376 (PMC4473034; doi:10.1371/journal.pone.0129376)
Supplement: S1 File — (Table A) Multivariate Multinomial Logistic Regression Model of Retention in Care and Viral Suppression, 2010–2011. (Table B) Multivariate Multinomial Logistic Regression Model of Retention in Care and Viral Suppression, 2010–2012. (DOCX) [file pone.0129376.s001.docx]

| **Characteristics** | **R/NS vs. R/S** | **NR/S vs. R/S** | **NR/NS vs. R/S** | **LTFU vs. R/S** |
| --- | --- | --- | --- | --- |
| **Status in 2010**  Retained/Suppressed  Retained/Not Suppressed  Not Retained/Suppressed  Not Retained/Not Suppressed | 1 [Reference]  **9.06 (7.94-10.32)**  1.26 (.967-1.64)  **7.16 (5.69-9.03)** | 1 [Reference]  1.26 (1.00-1.60)  **3.15 (2.63-3.78)**  **3.41 (2.48-4.68**) | 1 [Reference]  **10.47 (8.26-13.27)**  **4.69 (3.35-6.58)**  **24.4 (18.11-32.89)** | 1 [Reference]  **3.2 (2.77-3.70)**  **8.40 (7.25-9.73)**  **18.6 (15.23-22.69)** |
| **Age (years) in 2010**  18-29  30-39  40-49  ≥ 50 | 1 [Reference]  **0.65 (0.53-0.81)**  **0.74 (0.61-0.91)**  **0.53 (0.43-0.65)** | 1 [Reference]  1.03 (0.80-1.33)  0.86 (0.68-1.09)  **0.65 (0.51-0.84)** | 1 [Reference]  **0.62 (0.47-0.82)**  **0.46 (0.35-0.60)**  **0.27 (0.20-0.38)** | 1 [Reference]  **0.64 (0.53-0.78)**  **0.57 (0.48-0.68)**  **0.49 (0.41-0.59)** |
| **Sex**  Male  Female | 1 [Reference]  1.06 (0.92-1.22) | 1 [Reference]  1.05 (0.87-1.25) | 1 [Reference]  **0.75 (0.59-0.95)** | 1 [Reference]  **0.82 (0.72-0.94)** |
| **Race/Ethnicity**  White  Black  Hispanic  Other/Unknown | 1 [Reference]  **1.29 (1.03-1.40)**  1.02 (0.86-1.21)  0.97 (0.65-1.44) | 1 [Reference]  0.86 (0.72-1.02)  **0.69 (0.57-0.83)**  1.27 (0.90-1.81) | 1 [Reference]  1.25 (0.98-1.59)  **0.60 (0.44-0.81)**  0.56 (0.25-1.24) | 1 [Reference]  0.88 (0.77-1.01)  **0.75 (0.64-0.87)**  1.27 (0.93-1.73) |
| **HIV Risk Factor**  MSM  HET  IDU  Other/Unknown | 1 [Reference]  **0.85 (0.73-0.99)**  1.06 (0.90-1.25)  0.98 (0.71-1.36) | 1 [Reference]  0.87 (0.72-1.05)  1.04 (0.84-1.29)  1.04 (0.69-1.56) | 1 [Reference]  **0.78 (0.61-0.99)**  1.21 (0.91-1.60)  1.09 (0.65-1.85) | 1 [Reference]  **0.81 (0.70-0.93)**  1.12 (0.96-1.31)  1.21 (0.90-1.64) |
| **Insurance in 2010**  Private  Medicaid  Medicare  Uninsured/Ryan White  Other/Missing | 1 [Reference]  1.19 (0.98-1.46)  1.24 (1.00-1.53)  0.94 (0.76-1.17)  0.78 (0.52-1.17) | 1 [Reference]  **0.90 (0.65-0.98)**  **0.72 (0.58-0.89)**  0.82 (0.66-1.02)  1.03 (0.72-1.48) | 1 [Reference]  1.09 (0.78-1.53)  0.98 (0.68-1.41)  0.86 (0.60-1.22)  1.26 (0.73-2.19) | 1 [Reference]  0.92 (0.77-1.10)  0.91 (0.75-1.11)  1.04 (0.86-1.25)  1.26 (0.93-1.69) |
| **First CD4 Count in 2010**  ≤ 200 cell/mm^3^  201-350 cell/mm^3^  351-500 cell/mm^3^  > 500 cell/mm^3^  Unknown | 1 [Reference]  **0.63 (0.53-0.73)**  **0.48 (0.40-0.57)**  **0.43 (0.37-0.51)**  0.63 (0.30-1.35) | 1 [Reference]  1.13 (0.90-1.43)  0.98 (0.78-1.23)  1.03 (0.83-1.27)  0.96 (0.36-2.54) | 1 [Reference]  **0.65 (0.51-0.84)**  **0.42 (0.32-0.54)**  **0.30 (0.23-0.39)**  0.85 (0.34-2.14) | 1 [Reference]  **0.71 (0.60-0.83)**  **0.61 (0.52-0.71)**  **0.53 (0.45-0.61)**  **0.44 (0.22-0.90)** |
| **Use of ART in 2010**  No  Yes | 1 [Reference]  **0.26 (0.21-0.31)** | 1 [Reference]  1.39 (0.94-2.06) | 1 [Reference]  **0.31 (0.24-0.41)** | 1 [Reference]  **0.39 (0.32-0.47)** |

**Supplemental Information File 1**

**Table A:** Multivariate Multinomial Logistic Regression Model of Retention in Care and Viral Suppression, 2010-2011

**Abbreviations:** ART, antiretroviral therapy; HET, heterosexual transmission; HIV, human immunodeficiency virus; IDU, injection drug use; MSM, men who have sex with men; R=retained; S=suppressed (virologically); NR=not retained; NS=not suppressed (virologically); LTFU=loss to follow-up.

**Note:** Entries are adjusted relative risk ratios, with 95% confidence interval

**Table B:** Multivariate Multinomial Logistic Regression Model of Retention in Care and Viral Suppression, 2010-2012

| **Characteristics** | **R/NS vs. R/S** | **NR/S vs. R/S** | **NR/NS vs. R/S** | **LTFU vs. R/S** |
| --- | --- | --- | --- | --- |
| **Status in 2010**  Retained/Suppressed  Retained/Not Suppressed  Not Retained/Suppressed  Not Retained/Not Suppressed | 1 [Reference]  **5.61 (4.86-6.47)**  **1.41 (1.07-1.84)**  **5.04 (3.96-6.42)** | 1 [Reference]  **1.34 (1.09-1.65)**  **3.35 (2.79-4.02)**  **2.97 (2.21-3.98)** | 1 [Reference]  **7.36 (5.72-9.46)**  **4.42 (3.12-6.27)**  **13.21 (9.50-18.37)** | 1 [Reference]  **2.83 (2.5-3.21)**  **5.81 (5.05-6.67)**  **10.40 (8.67-12.47)** |
| **Age (years) in 2010**  18-29  30-39  40-49  ≥ 50 | 1 [Reference]  0.83 (0.67-1.04)  **0.69 (0.56-0.85)**  **0.51 (0.41-0.64)** | 1 [Reference]  **0.78 (0.61-0.97)**  **0.56 (0.45-0.70)**  **0.44 (0.35-0.56)** | 1 [Reference]  **0.65 (0.47-0.87)**  **0.39 (0.29-0.52)**  **0.25 (0.18-0.35)** | 1 [Reference]  **0.73 (0.62-0.86)**  **0.51 (0.44-0.60)**  **0.43 (0.36-0.50)** |
| **Sex**  Male  Female | 1 [Reference]  0.95 (0.82-1.10) | 1 [Reference]  **0.79 (0.66-0.94)** | 1 [Reference]  **0.70 (0.54-0.91)** | 1 [Reference]  **0.78 (0.69-0.88)** |
| **Race/Ethnicity**  White  Black  Hispanic  Other/Unknown | 1 [Reference]  1.05 (0.89-1.24)  **0.78 (0.65-0.94)**  1.17 (0.79-1.75) | 1 [Reference]  0.99 (0.83-1.16)  **0.71 (0.59-0.85)**  0.75 (0.49-1.14) | 1 [Reference]  0.86 (0.66-1.11)  **0.50 (0.36-0.68)**  0.72 (0.35-1.47) | 1 [Reference]  **0.79 (0.70-0.89)**  **0.66 (0.58-0.76)**  1.10 (0.84-1.45) |
| **HIV Risk Factor**  MSM  HET  IDU  Other/Unknown | 1 [Reference]  **0.77 (0.65-0.90)**  1.11 (0.93-1.33)  1.03 (0.73-1.46) | 1 [Reference]  0.85 (0.72-1.02)  1.00 (0.81-1.23)  0.93 (0.63-1.39) | 1 [Reference]  **0.76 (0.58-0.99)**  1.27 (0.94-1.73)  0.93 (0.51-1.70) | 1 [Reference]  **0.83 (0.74-0.94)**  **1.16 (1.01-1.33)**  1.14 (0.87-1.50) |
| **Insurance in 2010**  Private  Medicaid  Medicare  Uninsured/Ryan White  Other/Missing | 1 [Reference]  **1.26 (1.02-1.57)**  1.14 (0.90-1.44)  1.00 (0.80-1.28)  0.85 (0.54-1.35) | 1 [Reference]  0.97 (0.71-1.06)  **0.75 (0.60-0.93)**  1.05 (0.85-1.30)  1.09 (0.75-1.58) | 1 [Reference]  1.22 (0.84-1.78)  1.14 (0.76-1.70)  1.19 (0.81-1.76)  1.15 (0.59-2.23) | 1 [Reference]  0.99 (0.85-1.16)  **0.82 (0.70-0.97)**  0.97 (0.83-1.15)  **1.39 (1.08-1.80)** |
| **First CD4 Count in 2010**  ≤ 200 cell/mm^3^  201-350 cell/mm^3^  351-500 cell/mm^3^  > 500 cell/mm^3^  Unknown | 1 [Reference]  **0.74 (0.62-0.88)**  **0.54 (0.45-0.65)**  **0.48 (0.40-0.57)**  0.99 (0.42-2.32) | 1 [Reference]  0.92 (0.74-1.15)  0.92 (0.74-1.14)  0.91 (0.75-1.11)  1.42 (0.56-3.61) | 1 [Reference]  0.86 (0.65-1.13)  **0.52 (0.39-0.70)**  **0.43 (0.33-0.58)**  0.33 (0.04-2.54) | 1 [Reference]  **0.70 (0.61-0.81)**  **0.60 (0.52-0.69)**  **0.54 (0.47-0.61)**  1.36 (0.74-2.52) |
| **Use of ART in 2010**  No  Yes | 1 [Reference]  **0.46 (0.37-0.56)** | 1 [Reference]  1.00 (0.75-1.35) | 1 [Reference]  **0.57 (0.42-0.77)** | 1 [Reference]  **0.58 (0.48-0.68)** |

**Abbreviations:** ART, antiretroviral therapy; HET, heterosexual transmission; HIV, human immunodeficiency virus; IDU, injection drug use; MSM, men who have sex with men; R=retained; S=suppressed (virologically); NR=not retained; NS=not suppressed (virologically); LTFU=loss to follow-up.

**Note:** Entries are adjusted relative risk ratios, with 95% confidence interval
